# Contemporary global perspectives of medical students on research during undergraduate medical education: a systematic literature review

**DOI:** 10.1080/10872981.2018.1537430

**Published:** 2018-10-29

**Authors:** C. Stone, G. Y. Dogbey, S. Klenzak, K. Van Fossen, B. Tan, G. D. Brannan

**Affiliations:** a School of Osteopathic Medicine, Campbell University, Lillington, NC, USA; b Graduate Medical Education Department, Cape Fear Valley Health System, Fayetteville, NC, USA

**Keywords:** Evidence-based medicine, physician-scientist, research, undergraduate medical education, global perspectives

## Abstract

**Background**: The need for increased expertise in evidence-based medicine and concerns about the decreasing numbers of physician-scientists have underscored the need for promoting and encouraging research in medical education. The critical shortage of physician-scientists has assumed a dimension demanding a coordinated global response. This systematic review examined the perceptions of medical students regarding research during undergraduate medical school from a global perspective.

**Methods**: Articles for this review were searched using PubMed, SCOPUS and Cochrane. Studies published within the last 10 years of the start date of the study that met specified criteria were included. Identified articles were initially screened by title as well as keywords and their abstracts were further screened to determine relevance. Full-text of screened articles were read for validation prior to inclusion.

**Results**: A total of 26 articles from the literature met the set criteria for final inclusion. Contents of the abstracts and corresponding full-text articles were analyzed for themes on the research perspectives of medical students. The themes derived comprised: research interest, physician-scientist decline and shortage, responses to physician-scientist shortage, curriculum issues, skills (motivation and self-efficacy), research needs, socioeconomic and cultural issues, and barriers.

**Conclusion**: Despite the wide variations in medical education systems worldwide, the perspectives of medical students on research in undergraduate medical education shared many common themes. Globally, medical students underscored the necessity and importance of research in medical education as reflected by many students reporting positive attitudes and interest in research endeavors. Moreover, a worldwide consensus emerged regarding the decline in the numbers of physician-scientists and the necessity for a reversal of that trend. Various barriers to research engagement and participation were highlighted.

## Introduction

Two main drivers necessitating research during medical education have been the need for increased evidence-based medical practice and bench-to-bedside translational research with physician-scientists/researchers driving this process [1–6]. Hence, increasingly, the need for research as a critical component of modern medical education has not only gained currency but also urgency [2,3]. During undergraduate medical education, research instruction and involvement often provides the first opportunity for a medical student to gain exposure to research and participate in it. Medical students who engage in research learn how to formulate cogent thoughts from the generation of research ideas through study implementation to dissemination. Furthermore, they would have applied the scientific process in what they did, what they found, and how to report and explain such findings with rigorous detail and accuracy [3,7]. Such early immersions can contribute to the development of lasting habits of scientific thought and profound dispositions toward critical thinking [2,3], a vital aptitude that enhances success in the practice of medicine and engenders a crucial mentality that deserves early nurturing [1–3]. The importance of evidence-based medicine has meant that physicians are expected to be skilled and knowledgeable in the use of research and scientific methods of enquiry as applied to medical practice.

Indeed, clinician literacy in research improves critical thinking in guiding clinical judgement – necessary ingredients for the effective application of evidence-based medicine [7]. The ability to competently locate and critically appraise the appropriate medical literature, interpret volumes of data, integrate, and translate those for use in clinical situations is invaluable to medical practice [7,8]. The marriage between the clinical competence of a physician and the independent literature-based clinical evidence for informed decision-making could spell the difference between life and death of patients under potentially time-dependent conditions [7,9]. Such skills underscore the importance of the acquisition of research competencies and its crossover to the practice of evidence-based medicine [1–3].

The other related factor that undergirds the need for intensification of clinical research in medical education has been the continued decline in the numbers of physician-scientists (also sometimes referred to as clinician-researchers). Many studies have unequivocally shown that the numbers of physician-scientists have been in decline for many years [2,4–6,10–12]. In 2003, the number of physicians involved primarily in research was 1.8% compared to 4.6% in 1985 [6]. Another study found a lower percentage of 1.6% in 2011 compared to 3.6% in 1982 [11]. These alarming statistics about the decrease in the number of physician-scientists have not abated since the over four decades when it first received topical attention [5,6]. Indeed, the decline in numbers and scarcity of physician-scientists have assumed a global dimension. It is no longer an isolated issue confined only to some specific geographical regions of the world, it has become a worrisome issue with a global reach that requires concerted attention for redress [2,4].

Despite the expressed need and importance of clinical research, research training during undergraduate medical education in many countries is still inadequate, uncoordinated, constrained, and riddled with many difficulties [1,13]. Research instruction has often been for fulfilling required competencies of professionalism and continuing education. Furthermore, in some other instances, research in medical education is used for purposes of satisfying accreditation requirements. Key questions would be how do medical students perceive research during undergraduate medical training? What are their attitudes toward research? Do they perceive or understand research competencies as a critical component of their evidence-based knowledge? Does research appeal to them for purposes of evidence-based practice or contemplation to pursue a physician-scientist career in the future? Hence, it is important to understand the dynamics of the two drivers of research perspectives of medical students during medical school through a global prism. However, no known study has synthesized the available literature to highlight the global scope of the problems and the perspectives that pertained to it.

This systematic literature review synthesized the contemporary global perspectives (comprising perceptions, attitudes, beliefs, and dispositions) of medical students regarding research during their undergraduate training. It examined the ways in which medical students shared similar or different perspectives toward research during undergraduate medical education within a global context. Specific factors associated with their research perspectives and those with interest in a physician-scientist career path were explored. A deliberate delineation was made for comparative and contrast purposes between the U.S. landscape relative to the rest of the world for deeper insights, understanding, and search for solutions of emergent issues.

## Methods

### Search

The articles were obtained from PubMed, Scopus, and Cochrane databases covering a 10-year timeframe going back from 9/29/2007 to 9/29/2017 when the study was started. This search window was necessitated by using the start date of the study as the cutoff point so that the search was stabilized while capturing contemporary trends on the topic with the preceding 10 years as a prism. The search included title, abstracts, and keywords of studies from the global medical education literature. The search terms/keywords or phrases used for this review were as follows: Research, medical students, physician-scientist, career choices, osteopathic medical students, osteopathic medicine, perceptions, attitudes, motivations, and interests. The Boolean Operators ‘or’ and ‘and’ were used to expand and narrow the searches to include all the pertinent publications within the period under review. The use of osteopathic medical students in the key-word search was deliberate to ensure that, as a minority part of medical education in the U.S., its perspectives were represented as much as possible in the final literature. Only articles published in English were included and the search was conducted following the Preferred Reporting Items for Systematic Review and Meta-Analysis (PRISMA) guidelines.

### Eligibility criteria

Studies were excluded if they: 1) were not written in English, 2) involved program evaluations such as changes in student perceptions post-implementation of a specific curriculum or educational program, 3) combined data of medical students with physician or with other non-medical degree populations without clear distinction of data, 4) were Meta-analyses and Systematic Reviews older than 10 years as well as literature that mixed domestic and international medical student populations and, 5) were published letters to an editor, special communications, and opinions not based on methodology, results, and conclusions of a study.

### Study selection and process

Using the eligibility criteria, the preliminary list of literature was compiled by CS, BT, and GB who read the titles and abstracts of articles that were returned by the keyword search and the original number was reduced to those that met the inclusion criteria (See Search Strategies in Appendix). The abstracts of the subset of studies that met the criteria were read in entirety to determine relevance by CS, BT, KVF, and SK. Upon reading of the abstracts, if relevance was suspected or in question, the entirety of the article was read to determine its inclusion/exclusion. GD provided a global oversight by coordinating the consensus process as an independent eye. Articles that were not fully agreed upon for inclusion were discussed in a series of consensus building meetings until a consensus among the authors was reached.

### Data analysis

A quantitative analysis of the data obtained was purposefully not performed. This was because of difficulty in functional comparisons due to the variety of methods used in the studies together with the variations in medical education systems globally. In this study, we gathered the following information: author, country, sampled population, perspective studied, methods, and outcomes. We adopted a non-linear, iterative process involving content analysis for themes until a saturation point was reached where no new themes emerged. Constant comparisons were made among the group of authors to reconcile opinions for a consensual conclusion on themes.

## Results

**Figure 1.**
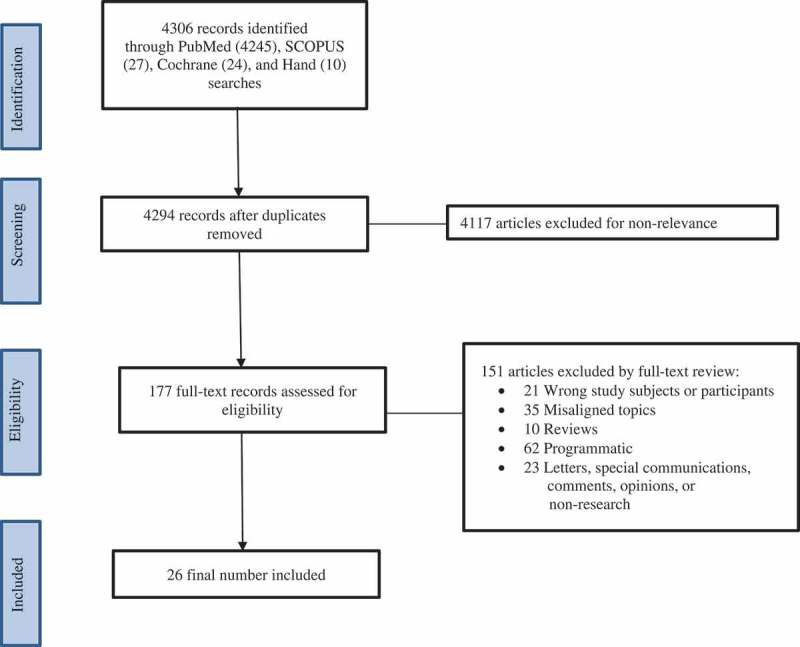
Schematic PRISMA diagram showing the literature selection process.

Figure 1 shows the schematic diagram of searching the literature. The title, abstract, and keywords, English language only, and the date of the search yielded 4306 studies. A further narrowing down yielded 4294 abstracts after removing duplicates. Furthermore, after excluding articles for non-relevance, 177 abstracts were obtained. The full-texts of these were further read in entirety resulting in the final inclusion of 26 studies. Upon reading of an abstract, if relevance was in question, the entirety of the article was read again by multiple researchers to determine if it tightly met inclusion/exclusion criteria.

The studies were from 13 countries representing different regions in the developed and developing worlds, (Table 1). Majority of the articles were from Pakistan and Saudi Arabia.

**Table 1.** Number of studies included by country/region of origin.

Country	Region	Number of Articles
Australia	Asia	1
Brazil	South America	3
Canada	North America	1
India	Asia	2
Iran	Middle East	1
Kuwait	Middle East	1
New Zealand	Asia	2
Pakistan	Asia	5
Saudi Arabia	Middle East	4
South Africa	Africa	1
Sweden	Europe	1
United States (U.S.)	North America	3
United Kingdom (U.K.)	Europe	1
TOTAL		26

Table 2 provides a bibliographic summary of the studies selected for the systematic review.

**Table 2.** Characteristics of each article included in the study.

*Source	Country Source	Sampled Population with N	Perspective Studied	Methods	Outcomes
Abu-Zaid et al. 2014 [54]	Saudi- Arabia	116/171 female medical students	Perceived barriers	-Online, self-rating survey, using Likert scale	-Top three barriers identified: Greater preference for patient care than research (75.0%); Work/life balance (52.6%); and lack of female role models (48.3%).
Abu-Zaid, 2014 [14]	Saudi-Arabia	61 female second year undergraduate medical students	Perceived attitudes	-Cross-sectional, self-rating survey	−31.1% had previous summer research experiences (68.0% local and 33.0% international programs). -All showed positive attitudes towards undergraduate research. - 72.0% endorsed the importance and 44.0% the compulsoriness of integrating scientific research into undergraduate medical education curricula.
Al-Halabi, 2014 [15]	Kuwait	150 sixth and seventh year medical students	Experiences, attitudes, and barriers	-Questionnaire	−66.0% of the students had participated in research. -54.0% students read medical journals regularly. -Barriers included: Lack of time due to other commitments or studies (33.9%), lack of interest either by the participant or other members of his/her project group (27.4%), lack of guidance and supervision (16.1%), lack of encouragement from the project’s mentor (12.1%), and lack of knowledge on how to write a scientific article for publication (10.5%). -42.0% of the students believed that research is important during undergraduate education, while 77.3% believed that it will become important during their future career.
Anbari et al. 2015 [58]	Iran	627 students in six schools of medical sciences	Barriers and infrastructure	-Quantitative and qualitative analytical approaches. - Research vs non-research groups -Barriers were validated using the Delphi technique on 36 students.	- Research students reported institutional barriers such as time, lack of access to electronic resources and prolongation of the process of buying equipment. -Non-research students reported individual barriers including lack of time, scientific writing skills, and access to trained assistants.
Baig, 2013 [33]	Pakistan	398 medical students at four medical universities	Perceptions, Prior experience and future research intent	-Data collection tool using a questionnaire and open-ended questions.	−88% intended to do research prior to medical school but only 51% reported they had previous research experience. -Reasons for engaging in research were to improve curriculum vitae (75%) and increase competitiveness for residency in the USA (43%).
Burgoyne, 2010 [16]	United Kingdom	317 undergraduate medical students	Transferable and research skills, prior research experience, attitude and motivation	-Questionnaire	−81% reported unawareness of opportunities. -51% expressed interest in research career. -56.5% reported moderate motivation, 35.8% high motivation, and 7.7% low motivation. -Barriers included: Isolation from friends (9%), felt like it was overly challenging or uninteresting (13%), undecided (11%).
Carter, 2016 [20]	United States (U.S)	266/346 osteopathic medical students	Research experience, interest during medical school	-Questionnaire	−81% had prior research experience. -75% had interest during medical school. -82% favored clinical research.
de Oliveira et al. 2011 [17]	Brazil	1004 medical students from 13 medical programs	-Availability and degree of involvement -Barriers	-Questionnaire	−7% had no interest in research. - 60% of them were involved in research training. -Barriers: Lack of institutional incentive, defective infrastructure, insufficient time available for professors to mentor undergraduate students.
Ejaz, 2011 [47]	Pakistan	387 final year medical students and fresh graduates	Research involvement, interest and barriers	-Cross-sectional questionnaire	-Medical students results only: 49% conducted literature search, 65% had journal reading experience due to the requirement of their institution. 41% undergraduates had already participated in a research project, however, mostly in the field as data collectors or computer assistants. -Barriers included: Current research status and teachings of Pakistan to be inadequate.
Funston et al. 2016 [48]	United Kingdom (UK)	1625 responses from 38 countries (excluding US); analysis restricted to countries with > 100 responses (n = 890)	Perceptions	-Online questionnaire	-Less than 50% thought their institution provided adequate research training. -Key barriers were lack of time and mentors. -Females were less interested. -The barriers and satisfaction with research training differed significantly among countries.
Jimmy, 2013 [29]	India	114 medical students	Perception and benefits	-Cross sectional study: Questionnaires	−20.9% had publications. -81.7% saw research being essential to understanding. -38.3% thought it cumbersome. -24.3% of the students were involved in research purely for acquiring knowledge. -18.3% were pursuing interest in research, while 1.7% were doing research to improve their curriculum vitae. -73.9% found it essential for all students to do research. -Barriers found: Lack of time, lack of incentives in the form of scholarships, and post-graduate specialty selection.
Kharraz et al. 2016 [55]	Saudi Arabia	221/350 medical students.	Perceived barriers	-Online, cross-sectional, self-rating survey	-Participation significantly differed by gender (males vs. females): 68.6% vs. 45.4%. -Top three barriers: Lack of time (77.4%), lack of formal courses in curriculum (76%), and lack of UR mentors (70.1%). Others were lack of mentors, lack of interest in research, lack of finding same-gender research mentor, and lack of opportunities.
Kumar, 2009 [40]	India	471 medical students	Awareness, perceptions and practices	-Questionnaire-based qualitative study	−70% were aware about research although the level of awareness varied. -Various skills of conducting research were known to 47% of the students. -76% were part of a research team mainly as a part of the medical curriculum. -8.3% were confident of research as a career option.
Mahmood, 2017 [4]	Pakistan	294 medical students	-Current research practices. -Future intentions. -Related motivations, barriers, and sought-after interventions	-Self-administered questionnaire	-Intentions to pursue research at a professional level remained low (19.7%). -Intentions decreased each passing year of study. -Most commonly expressed motivation for pursuing research was “admission into a residency program” (71.8%). -Such intention was associated with a decreased likelihood of pursing research professionally. -Barriers: Lack of time (72.4%), lack of supervisors (50.3%), lack of opportunity (48.3%).
Meraj, 2016 [34]	Pakistan	172 medical students	Perceptions and attitudes	-Cross-sectional questionnaire	−45.3% were aware of research opportunities. -65.7% thought research was important to future career and relevant to their lives -41.9% not interested in research as a career. -41.3% students enjoyed research -70% perceived research as stressful and 62.2% complex
Mina et al., 2016 [32]	Saudi Arabia	218/350 medical students	Perception and participation	-Online survey	-Top three influential factors: Facilitate entry into competitive residency programs (88.1%), improve curriculum vitae (81.2%), and publish in peer-reviewed journals (79.8%). -Participation in research significantly differed by gender, academic year, and GPA.
Moraes, 2016 [27]	Brazil	278 medical students from first to sixth year.	Interest in research	-Cross-sectional, self-administered questionnaire	−81.7% were interested in research. -Only 4.7% of the students thought research is important. -No statistically significant association with age, gender, number of physicians in their family, prior college courses were found.
Nel et al., 2013 [30]	South Africa	733 medical students	Attitudes	-Cross-sectional survey	−61% had positive attitudes toward research. -74% thought involvement is important -22% voluntarily engaged in research -4% presented at a meeting and 3% had published Perceived barriers: Lack of adequate training, time, and research opportunities.
Oliveira, 2014 [49]	Brazil	415 first through sixth year medical students	Understanding, advantages, difficulties and motivations	-Questionnaire	−47.2% were involved in research. -Main barrier reported was time constraint. -Among students not involved in research, 91.1% reported, however, that they favored its inclusion in the curriculum.
O’ Sullivan, 2009 [50]	United States (U.S)	40 medical students	Attitudes	-Qualitative based on interviews conducted in person at locations chosen by participant.	- Five (5) themes were reported: Early exposure to research, role models and mentoring, career pathways, interplay of personal and social factors, and career support for junior faculty members.
Park et al., 2010 [35]	New Zealand	558 medical students	Attitudes	-Questionnaire	−25% participated in some form of research activity, mostly in the summer, during medical school. -70% expressed interest in participating in research during medical school. -68% of respondents were aware of the intercalated research degree option but only 8.6% were interested. -35% of respondents planned to be involved in research throughout their medical career. -More students rated lifestyle (84% affirmative) and earning potential (43% affirmative) as more important factors than opportunity for research (23% affirmative) when choosing a career specialty.
Pathipati, 2016 [31]	United States (U.S)	328 medical students	Perception of research year	-Online survey at 5 medical schools with highly regarded research programs	-Reasons for research years off: Increase competitiveness for residency application (32%), time to pursue other opportunities (24%), and academic interest (23%). -Students who would still take a research year even if they were already assured a position in a residency program of their choice were at 65%, while 35% would not take a research year.
Rosenkranz, 2015 [18]	Australia	579/806 medical students from first year through fifth year	Attitudes, motivations, and barriers	-Convergent parallel mixed methods study using cross-sectional quantitative survey data and qualitative semi-structured interview findings	−7.5 % of students had prior research experience. -42% indicated interest beyond medical school. -89.1% of students believe conducting research is advantageous for medical career. -44.2% believe bureaucracy surrounding research is a significant deterrent. -49.8% of students were neutral to research and lower salary.
Sheikh, 2013 [59]	Pakistan	122 students	Interest and enthusiasm	-Case (not interested in research)-control (those interested) study	-Barriers identified by interested students: Curriculum overload, internet inexperience, an uncooperative community, difficulty in finding a mentor and selecting a topic, lack of previous exposure, and lack of internet facilities. -Barriers identified by non-interested students: sleep loss, extracurricular activities, uncooperative colleagues, inclement weather (hot and humid), lack of knowledge, bad past experiences, social commitments, drugs/addictions, laziness, uncooperative faculty, transportation problems, lack of motives and incentives, faculty-forced research, fatigue, and an attitude that considered research useless.
Siemens, 2010 [51]	Canada	327 second and fourth year medical students at three medical schools.	Attitudes and experience	-Questionnaire	-Involved in research prior to medical school (87%). -43% were not significantly involved in research during medical school. -24% were not interested. -44% believed research is important in future career. -Barriers included: Time, availability of research mentors, lack of formal teaching of research methodology, and lack of appropriate acknowledgement.
Stockfelt, 2016 [19]	Sweden	471 medical students	Research interest and activity	-Questionnaire administered as a follow up to a 2006 survey	−16% were actively involved in research. -36% were interested. -Barriers to research included: Lack of time (23%), excessive workload (22%), and not enough time to study (16%). -Financial compensation, expansion of research opportunities, and career planning would aid in research interest.

*Brackets denotes the number in the reference list.

Table 3 lists the emergent themes that characterized the global perspectives of medical students toward research during medical school.

**Table 3.** Common themes from global perspectives on research during medical education derived from content analysis of the abstracts/full-text articles.

Theme	Perspectives
Research interest	-Medical students express very high interest in research while in training. -Majority have positive attitude toward research.
Physician-scientist career (decline and shortage)	-Universal consensus on the reality of a decreasing numbers of physician-scientists that should not be ignored.
Response to physician-scientist shortage – MD/PhD and other combined programs, Specialized programs, NGOs, Curriculum	-Dual degrees MD/DO/PhD. -Curriculum changes reflecting research and academic medicine tracks. -Intercalated programs (Equivalence of dual degrees). -Research courses or fairs featuring keynote speakers, presentations, etc. (Germany). -Development of national research programs (Norway). -Voluntary immersion at individual institutions (Lebanon). -Workshops (Saudi Arabia).
Curriculum	-Curriculum often did not incorporate research across the board. -Curriculum not adequate in focusing on research in medical education. -No curriculum at all in some other situations. -Research was buried in the curriculum and not obvious. -Curriculum efforts varied greatly across institutions.
Skills – Motivation, Self-efficacy	-Willing to do research but lack the necessary skills and self-efficacy. -Well motivated but constrained by barriers. -Lack the necessary skills by little exposure to research earlier. -Highly motivated but not self-efficacious.
Needs – Training, Curriculum, Infrastructure, Competitiveness for residency/fellowships for residents	-Curriculum-based training in research necessary to provide the basic skills in research. -Training in clinical research. -Availability of research infrastructure. -Research itself needed for competitiveness for fellowships and residencies for students. -Needs research to improve CV. -For getting into academic medicine or becoming a physician-scientist.
Socio-Economic and Cultural – Organization, Gender	-Males rather than females would want to go into academic medicine or physician-scientists than females. -Cultural issues in some countries where the two genders do not mix. -Males more likely to engage in research than females.
Barriers – Institutional, Non-institutional	-Institutional barriers such as time unavailability, lack of mentors, inadequate support for research, lack of access to electronic resources, lack of mentors, and prolongation of the process of buying equipment, and lack of infrastructure. -Non-institutional: Lack of time, inadequate scientific writing skills, lack of early exposure to research and lack of access to assistance.

These were derived from the 26 studies that were finally included as the data for the review. These themes were common across the studies within the medical education contexts of the U.S. and other countries outside the U.S. The themes derived were as follows: Research interest, physician-scientist decline and shortage, responses to the physician-scientist shortage, curriculum issues, skills (motivation and self-efficacy), research needs, socioeconomic and cultural issues, and barriers.

## Discussion

Out of the 26 selected articles, 23 (89%) were from countries outside the U.S. and 3 (11%) from the U.S. One plausible explanation of this result could be that in the U.S., most studies involved the assessment (pre-post) of specific programs involving research in medical education and therefore were excluded according to the study inclusion/exclusion criteria. Another possible explanation is that the physician-scientist shortage in the U.S. had been recognized decades earlier following the 1979 seminal work of Wyngaarden [11]. Ever since, the issue has been studied for decades, hence most of the perspectives were likely reported in articles published earlier than our timeframe considered in the inclusion criteria for this present study. This could indicate a positive direction in the sense that programs in the U.S. have been trying to address this problem over many decades. Moreover, the U.S. literature was replete with programmatic endeavors directed at fostering training of research savvy physicians and addressing the physician-scientist shortage.

By the exclusion criteria of this study, studies with programmatic leanings were excluded. One exception to note regarding the U.S. medical education landscape, however, is that exposure of osteopathic (DO) medical students to research is still in an early growth phase with limited data compared to their allopathic (MD) counterparts [20] hence its relative over representation in the U.S. literature in this current study. According to the National Resident Matching Program match data, U.S. allopathic students lead in the number of research experiences, followed by non-U.S. international medical graduates (IMG), U.S. IMG’s, and then osteopathic medical students [21–23]. Although osteopathic medical research has been on the rise [24], historically there has been a lack of culture promoting research [25] and even today, the osteopathic medical field still lags the allopathic field in the conduct of clinical research [26].

This current systematic review derived common themes that threaded through medical students’ research perspectives globally. Broad consensus was found on these themes with minor context-specific and geographic variations.

### Research interest

One common strand that was found in the perspectives of research during medical education was the medical students’ expressed interest and positive attitude toward research [20,27,28]. Overall, medical students viewed research as useful and important to their education [29,30]. However, motivations behind this interest varied from context to context across countries. For example, some students were interested in research to be competitive for residency [4,31,32], enhance curriculum vitae or resumés [32,33], and for some international medical students to gain admission into U.S. residency programs [33]. Nonetheless, the expressed interest in research did not translate into many more medical students thinking of becoming physician-scientists. Moreover, many of such students did not indicate if research engagement was to help them engage in future evidence-based practice or pursue a research career [4,34].

### Physician-scientist: decreasing numbers and shortage

There was a near universal accord regarding the recognition of the decline and shortage of physician-scientists as a problem that demanded serious attention. The literature was replete with expressed concerns about the lack of enthusiasm on the part of budding physicians to pursue the physician-scientist career track. Indeed, although many medical students indicated the importance of research in their education, they were not favorably inclined to pursue it as a career [4]. Various reasons were adduced for the lack of interest in a clinical research career path. Some of those reasons were preference for direct patient care as a clinician, lifestyle of a physician rather than a physician-scientist, diminished desire in prolonged training and accrual of extra debt, need to pay-off current student debt, difficulty in seeking extra-mural funds, lack of mentorship, and lack of interest in a scientific/research career [4–6].

### Response to physician-scientist shortage

Recognition of the decreasing numbers of physician-scientists as well as shortages led to the adoption of measures to counter or arrest the decline. Although we excluded program-specific studies from this review, it is important to point out that some of the studies that met the inclusion criteria suggested solutions to this problem. Some of those included dual-degrees such as MD/PhD or intercalated programs (as they are referred to in some countries outside the U.S [35]. Other combined programs and specialized programs were introduced by various medical colleges especially in the U.S. and Canada [36].

Some of the responses were premised on the idea that medical students should be equipped with research skills. To address student needs, a multitude of solutions from different countries have been experimented to expose medical students to research, encourage participation, and develop the requisite skills to be successful. Some examples include the development of national research programs (Norway) [37], voluntary immersion at individual institutions (Lebanon) [38], workshops (Saudi Arabia) [39] (India) [40], research courses (U.S.) [41], or fairs featuring keynote speakers, presentations, (Germany) [42], all of which have been met with varying degrees of success.

The most common reasons for not pursuing a dual or intercalated degree option were lack of interest, social reasons, and financial reasons [28,35]. There was no widespread support from the students for having research training as a compulsory part of medical school curriculum. With respect to long-term career plans, just a small proportion reported interest in wanting to become physician-scientists [34]. However, more students rated lifestyle and earning potential as more important factors than opportunity for research when choosing a career specialty [34,35].

### Curriculum

There was a high-level of agreement in the literature among medical students that despite the importance of research, it often was not well represented in their curricula. Curricula were often overloaded with basic and clinical science subjects with little room for research instruction and learning. In most cases, research participation was an extra-curricular activity where students were not well prepared in the basic concepts of epidemiology, statistics, and scientific investigation [41,43]. Indeed, students often reported low confidence in understanding medical literature and statistical analysis. Windish et al. [44], for example, surveyed 11 residency programs in the U.S. to assess biostatistics and research interpretation – the result was an average of 40% score on a multiple-choice test. Such a finding highlighted that knowledge deficits in research competencies and skills during medical school often spilled over to residency. This result was consistent with the literature elsewhere within the global context [44–46]. Curriculum that incorporates, enacts, and implements research during undergraduate medical education is critical because students who got trained and involved in research were more likely to continue to do research in the future [36].

### Skills

Many medical students lacked the skills to pursue research during undergraduate medical education [47,49]. This lack of skills may arise from inadequate early exposure to the basic concepts of scientific inquiry that serves to limit their later research involvement [49–51]. While in some countries students had research exposure before medical school, they still did not feel prepared enough to engage in research [35,47,50]. A plausible explanation could be that the previous research experience was not targeted enough to facilitate their participation in applied research during medical school. This lack of skills perhaps resulted in low motivation and lack of self-efficacy among many students [47].

### Research needs

Medical students worldwide expressed needs that, if addressed, would facilitate their participation and engagement in research during medical school. These needs included, mainly but not exclusively, research training, curriculum enhancements, time availability, and availability of infrastructure for research. Indeed, these needs coincided with some of the other themes that were gleaned from the review. Some institutions accommodate students by allowing research to be conducted during their spare time using technology [46], or through the implementation of a more flexible preclinical curriculum [52].

### Socio-economic and cultural

Socio-economic factors play a substantial role in undergraduate medical education and could shape students reasoning and aspirations for pursuing research careers as physician-scientists. For some students outside the U.S., for instance, the motivation to participate in research was to boost their curriculum vitae (CVs) to allow for their career advancement and pursuing opportunities in developed countries [53]. Similarly, students in developed countries who engage in research are often motivated by the goal of getting into a more competitive residency program. In other regions of the world, particularly in certain countries such as Saudi Arabia, Pakistan, and Iran, cultural and religious contexts interplay with research during undergraduate medical education – this was quite a unique theme relevant to these settings. For instance, lack of mentors for female students was due to religious gender restrictions where male clinicians could not mentor female students [1,54,55] in some circumstances.

### Barriers – institutional, non-institutional

Despite the varied approaches and efforts at improving research during medical school, the literature amply provided instances of students expressing both institutional and non-institutional barriers to successful research participation. Prevalent institutional barriers included limited time, lack of access to electronic resources, lack of mentors, lack of supporting infrastructure, and prolongation of the process of acquiring equipment. Non-institutional barriers included lack of down time, inadequate scientific writing skills, and lack of self-efficacy. Many of the studies [51,56] that investigated factors working against student involvement in undergraduate research cited other key factors such as poor mentorship, lack of role models, and perceived lower salaries of academic physicians. Indeed, quality mentorship is strongly associated with a more positive research experience for students and may bolster interest in a research-oriented career [51,56].

While there exists a considerable overlap regarding contemporary perceptions of barriers toward research globally, students from developing countries face many more hurdles not only to their research participation but medical education in general. Examples include lack of funds [57]), insufficient access to electronic resources [58], constrained availability of the internet [57,59] and sometimes, even war/conflicts [57].

Several limitations to this review should be noted. As mentioned earlier, our search excluded many U.S. articles by our inclusion criteria. Moreover, only studies written in English were included. There was also difficulty in functional comparisons as there were a variety in the details of the methods used in the studies and variations in medical education systems globally.

## Conclusion and recommendation

This review examined the perspectives of medical students regarding research during medical education from a global perspective. Increasingly, research in medical education has been encouraged throughout the continuum of medical education. While these programs and efforts are laudable, they have not been without controversies. Outcomes have been mixed and the targets (medical students) were sometimes unaware of the opportunities available to them. Some medical students also engaged or participated in research for reasons other than for evidence-based practice or pursuit of physician-scientist career.

A consensus of medical students regarding the need and importance of research in undergraduate medical education exists in a global context. While the need and importance of research were upheld, barriers to it were passionately expressed. The perspective on the decline in the numbers of physician-scientists was that of a global accord. Differences in the thematic perspectives of research in medical education were quite minor and difficult to finely characterize.

Medical education systems vary greatly worldwide yet medical student perspectives on research in medical education revealed several common universal themes. Hence, a convergence of ideas amongst medical educators from different cultures could be possible to enhance research in undergraduate medical education. A global effort that harnesses an understanding of the concordances in the perspectives on the issues faced by medical students regarding research instruction and learning in medical schools could foster the training of physicians imbued with evidence-based practice and perhaps more inclined to become physician-scientists. Thus, a coordinated global response could promote and re-emphasize the importance of evidence-based medicine in clinical practice and potentially address the decline in the numbers of physician-scientists.

## Appendix

### Search Strategy

**Table:**

Database		Strategy/Limits
PubMed	1	Medical student* OR students, medical [MeSH] OR osteopathic medical student* OR osteopathic medicine[MeSH]
	2	Research OR research[MeSH] OR career choice* OR career choice[MeSH] OR physician scientist*
	3	1 AND 2
	4	Motivation* OR motivation[MeSH] OR attitude* OR attitude[MeSH] OR perception* OR perception[MeSH] OR interest
	5	3 AND 4 (Limited to 9/29/2007–9/29/2017
Database		Strategy/Limits
Scopus	1	[TITLE-ABS-KEY] ‘medical student’ OR ‘medical students’ OR ‘osteopathic medical student’ OR ‘osteopathic medical students’ OR ‘osteopathic medicine’
	2	[TITLE-ABS-KEY] ‘career choice’ OR ‘career choices’ OR career OR careers
	3	[TITLE-ABS-KEY] research
	4	[TITLE-ABS-KEY] ‘physician scientist’ OR ‘physician scientists’
	5	[TITLE-ABS-KEY] motivation OR motivations OR attitude OR attitudes OR perceptions OR perception OR interest OR interests
	6	1 AND 2 AND 3 AND 4 AND 5
	7	PUBYEAR limited to 2007–2017, LANG English
Database		Strategy/Limits
Cochrane	#1	medical students
	#2	MeSH descriptor: [Students, Medical] explode all trees
	#3	#1 or #2
	#4	osteopathic medical students
	#5	osteopathic medicine
	#6	MeSH descriptor: [Osteopathic Medicine] explode all trees
	#7	#4 or #5 or #6
	#8	#7 or #3
	#9	perceptions
	#10	motivations
	#11	attitudes
	#12	interests
	#13	MeSH descriptor: [Motivation] explode all trees
	#14	MeSH descriptor: [Perception] explode all trees
	#15	MeSH descriptor: [Attitude] explode all trees
	#16	#9 or #10 or #11 or #12 or #13 or #14 or #15
	#17	career
	#18	career choice
	#19	MeSH descriptor: [Career Choice] explode all trees
	#20	#17 or #18 or #19
	#21	physician scientist
	#22	physician researcher
	#23	#21 or #22
	#24	#3 or #7
	#25	#24 and #16
	#26	#20 and #25
	#27	#23 and #26
	#28	#8 and #16
	#29	#20 and #28
	#30	#29 and #23
	#31	research
	#32	MeSH descriptor: [Research] explode all trees
	#33	#31 or #32
	#34	#30 and #33 Publication Year from 2007 to 2017
